# Melanocortin-4 Receptor Mutations and Polymorphisms Do Not Affect Weight Loss after Bariatric Surgery

**DOI:** 10.1371/journal.pone.0048221

**Published:** 2012-11-21

**Authors:** Marion Valette, Christine Poitou, Johanne Le Beyec, Jean-Luc Bouillot, Karine Clement, Sébastien Czernichow

**Affiliations:** 1 Paris 13 University, Sorbone Paris Cité, Nutritional Epidemiology Research Unit-UMR U557 INSERM, U1125 INRA, CNAM, CRNH-IdF, Bobigny, France; 2 Assistance Publique Hopitaux de Paris, Heart and Metabolism Department, Pitié Salpêtrière Hospital, Paris, France; 3 Institut National de la Santé et de la Recherche Médicale, U872 Team7, Nutriomique, Cordelier Research Center, Paris, France; 4 Pierre et Marie Curie University, Paris, France; 5 Assistance Publique Hopitaux de Paris, Nutrigenic Unit, Endocrinology and Oncology Biochemistry Department, Pitié-Salpêtrière Hospital, Paris, France; 6 APHP, Department Medical-Surgical, Ambroise Paré Hospital, Boulogne-Billancourt, France; 7 University of Versailles Saint Quentin en Yvelines, Boulogne-Billancourt, France; 8 INSERM U1018, Centre for Research in Epidemiology and Population Health, Villejuif, France; University of Texas Health Science Center at San Antonio, United States of America

## Abstract

Bariatric surgery is the most effective long term weight-loss therapy for severe and morbidly obese patients. Melanocortin-4 Receptor (MC4R) mutations, the most frequent known cause of monogenic obesity, affect the regulation of energy homeostasis. The impact of such mutations on weight loss after bariatric surgery is still debated.

The objective is to determine the impact of MC4R status on weight loss in obese subjects over one year after bariatric surgery.

A total of 648 patients, who were referred to bariatric surgery in a single clinical nutrition department, were genotyped for their MC4R status. The following four groups were categorized: functional *MC4R* mutations, *MC4R* single nucleotide polymorphisms (SNPs): Val103Ile (V103L) and Ile251Leu (I251L), *MC4R* variant *rs17782313* (downstream of *MC4R*) and *MC4R* SNP A-178C on the promoter. Each patient was matched with two randomly paired controls without mutation. Matching factors were age, sex, baseline weight and type of surgery procedure (Roux-en-Y gastric bypass and adjustable gastric banding). We compared weight loss between cases and controls at 3, 6 and 12 months after surgery.

Among 648 patients, we identified 9 carriers of functional *MC4R* mutations, 10 carriers of *MC4R* V103L and I251L SNPs, 7 carriers of the rs17792313 variant and 22 carriers of the A-178C SNP. Weight loss at 3, 6 and 12 months did not differ between cases and controls, whatever the *MC4R* mutations.

This is the first case-control study to show that MC4R mutations and polymorphisms do not affect weight loss and body composition over one year after bariatric surgery.

## Introduction

Obesity is a rapidly growing global public health challenge [1]. It is directly related to an increased risk for type 2 diabetes, hypertension, dyslipidemia, cardiovascular disease and overall mortality [2]. Bariatric surgery is the most effective therapy for long-term weight lost in severe or morbidly obese subjects [3], [4]. Its use has dramatically increased during the last decade [1]. However, the individual weight loss response varies widely. Clinical and genetic factors may influence its effect. Genetic background accounts for 40% to 70% of an individual predisposition to obesity [5] with strong determinants of weight loss following bariatric surgery [6].

Melanocortin-4 receptor (MC4R) mutations are the most frequent monogenic causes of severe early onset human obesity (up to 6%) [7]. MC4R expressed in the hypothalamic nuclei is involved in the leptin-melanocortin signaling pathways. The central melanocortin system influences energy intake and expenditure and the balance between lipid utilization and storage. MC4R affects leptin-melanocortin signaling system and regulates food intake and maintain long-term energy homeostasis by integrating signals provided by its agonist, α-melanocyte stimulating hormone, and antagonist, Agouti related peptide [8]. By this way, *MC4R* mutations may play a role in weight loss, especially after bariatric surgery.

Several *MC4R* mutations affect differently this system. Partial or total loss of MC4R function, as well as the variant rs17782313 mapped 188 kb downstream of *MC4R*, are positively associated with obesity [9]–[11]. In contrast, two SNPs of the MC4R gene: I251L and V103L have been shown to be negatively correlated with obesity [12], [13]. Promoter mutations could be associated with obesity [14], although the effect of the polymorphism A-178C was not specifically investigated.

We tested whether these deoxyribose nucleic acid (DNA) sequence variations in and downstream of the MC4R gene affects weight loss and body composition after the two most frequent bariatric procedures: Roux-en-Y gastric bypass (RYGBP) and adjustable gastric banding (AGB) [15].

## Materials and Methods

### Subjects

Subjects were patients followed-up in the Department of Nutrition of Pitié-Salpêtrière hospital (Paris, France). This study enrolled 648 severely obese adults with a body mass index (BMI) >35 kg/m^2^ who underwent primary bariatric surgery from 1998 until 2010. Two laparoscopic procedures (AGB and RYGBP) [15], were performed by the same surgical team (J.L. Bouillot) in the Department of Surgery of Hotel-Dieu hospital. All patients underwent a preoperative assessment and were also examined at 3, 6 and 12 months after surgery. The clinical investigation was approved by the Ethics Committee of Hotel-Dieu Hospital. All subjects gave their written informed consent.

### Anthropometric and biochemical measurements

Anthropometric parameters, including BMI and fat mass determined by biphotonic absorptiometry DEXA (dual-energy x-ray absorptiometry; Hologic, Waltham, MA) were assessed before and at 3, 6 and 12 months after surgery. Height was obtained at the initial visit and weight measurement obtained at each visit. BMI was calculated as the weight (kilograms) divided by the square of the height (square meters). Blood samples for DNA extraction were collected on EDTA after an overnight fast at baseline. Biochemical parameters including fasting glycemia and total cholesterol were measured using routine techniques.

### Blood sampling and genomic sequencing

Genomic DNA was extracted from peripheral blood according to standard methods (ExtraGene extractor (Genomic SA) using Wizard Genomic DNA purification kit (Promega). Screening for *MC4R* variants and mutations was performed by direct sequencing of four PCR products (primers sequences available on demand) mapping the whole *MC4R* coding sequence as well as 230pb downstream of the *ATG* site (ABI 3730 sequencer, Applied Biosystems, Foster City, USA). Sequence data were aligned using the Seqscape 2.5 software and compared to the *MC4R* sequence (NM_005912.2). In *silico* prediction of the deleterious effect of the mutant was performed with the Alamut Mutation Interpretation Software (Interactive Biosoftware, Rouen, France) and the PolyPhen Web site (http://genetics bwh.harvard.edu/pph). Among the various MC4R variants studied in this work, two news mutations (Cys196Tyr, Leu134Pro) had been deposited in GeneBank. Their publications are in process and their accession numbers are JX515607 and JX51608.

### Study design and statistical analysis

Patients were categorized as carriers of: functional *MC4R* mutations, V103L and I251L SNPs, *rs17782313* variant or SNP A-178C. Each case was matched with two randomly paired controls on the following covariates: age, sex, baseline weight, surgery procedure. Difference in weight change between case and their matched controls of each group was computed at 3, 6 and 12 months after surgery. Weight was expressed as a percentage of body weight in kilograms at the times of surgery. Fat mass as a percentage of weight (FM %) change was calculated at baseline and at 12 months. Wilcoxon signed rank tests were used to compare mean differences between cases and controls at each time of follow-up. All statistical analyses were performed with SAS version 9.1 (SAS institute, Cary, NC, USA).

## Results

### *MC4R* genotyping

Among the 648 patients screened for *MC4R* mutations, 9 were heterozygous for functional *MC4R* mutations, 10 heterozygous for *MC4R* SNPs V103L and I251L, 22 were heterozygous for the SNP A-178C located on the *MC4R* promoter and 7 carriers (4 heterozygous and 3 homozygous) of the variant rs17782313 located downstream of *MC4R*. Baseline characteristics are shown in Table 1 and indicate no difference between groups in fasting blood glucose, cholesterolemia and others characteristics, except for a modest difference for age in A-178C SNP carriers compared to their controls. Moreover, fasting blood glucose and cholesterolemia did not differ 12 months after surgery between case and controls (data not presented).

**Table 1:** Clinical characteristics according to MC4R genotypes (functional *MC4R* mutations, MC4R polymorphisms and the variant rs17782313 downstream *MC4R*).

		Functional MC4R mutations		Variant rs17782313		MC4R polymorphisms: Val130Ile, Ile251Leu		MC4R polymorphism: A-178C	
		Carriers	Non carriers	Carriers	Non carriers	Carriers	Non carriers	Carriers	Non carriers
Number, n		9	18	7	14	10	20	22	44
Age, years		36.2 (13.3)	34.2 (8.4)	45.4 (9.2)	46.1(9.9)	39.2 (12.2)	42.4 (9.1)	46.1 (7.9)*	43.3 (9.1)*
% Female		100	100	71.4	71.4	80	80	86.4	86.4
Surgery Type	Gastric banding	3	6	0	0	2	4	0	0
	Bypass	6	12	7	14	8	16	22	44
Weight, kg	Baseline	139.2 (14.3)	139.8 (12.1)	142.0 (23.9)	133.4 (15.5)	121.6 (18.2)	125.1 (16.0)	126.4 (16.4)	124.3 (13.9)
BMI, kg/m^2^	Baseline	50.4 (4.6)	50.8 (4.9)	49.0 (7.6)	48.8 (6.3)	46.0 (6.1)	46.0 (5.6)	46.1 (6.4)	46.1 (5.3)
Fat mass, %	Baseline	47.9 (4.1)	49.7 (4.9)	45.3 (4.3)	46.3 (5.5)	45.5 (4.3)	45.1 (7.6)	45.3 (4.9)	45.6 (6.2)
Glycemia, mmol/L	Baseline	5.1 (1.0)	6.0 (2.2)	7.2 (2.7)	6.3 (1.7)	5.5 (1.3)	6.1 (2.9)	6.1 (1.7)	6.4 (2.8)
Cholesterol, mmol/L	Baseline	5.0 (1.3)	4.8 (0.8)	4.5 (0.8)	4.5 (0.8)	5.2 (1.0)	5.0 (0.9)	4.9 (0.8)	5.1 (1.3)
Percentage of Weight, %	3 months	84.9 (6.5)	84.1 (6.8)	83.1 (7.6)	81.3 (4.3)	84.8 (4.6)	83.3 (6.8)	83.0 (3.7)	82.4 (4.3)
	6 months	80.0 (10)	78.8 (9.2)	76.1 (10.6)	74.4 (5.6)	78.9 (7.2)	77.3 (8.3)	76.5 (6.2)	74.5 (5.9)
	12 months	74.1 (13.4)	72.5 (10.4)	70.9 (13.3)	68.0 (7.1)	74.3 (9.5)	72.4 (10.5)	70.2 (7.9)	68.7 (6.8)

Data are mean (SD) or % when specified.

Difference between carriers and non-carriers were tested with Wilcoxon paired rank test.

* p value<0.05.

### Baseline and post-surgery weight loss and body composition

The A-178C carrier group showed a difference in weight at 6 months which was not maintained. At 6 months of follow-up, weight was significantly higher for polymorphism A-178C carriers (96.6±14.4 kg) compared to non-carrier (92.4±10.8 kg), but this difference disappeared 12 months after surgery. However, this comparison did not show any difference when weight expressed as a percentage of body weight were used instead of weight.

**Figure 1:**
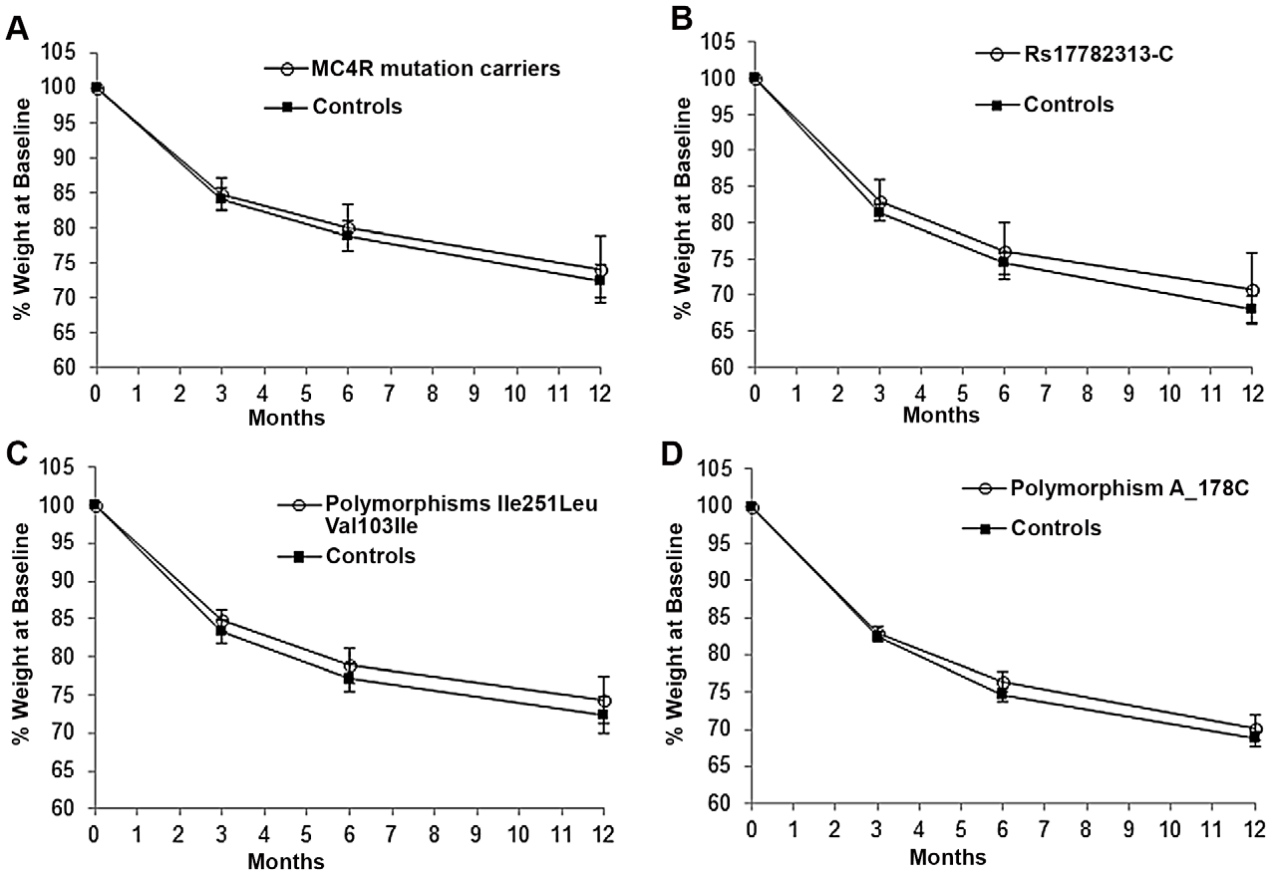
Weight loss over 12 months after bariatric surgery according to MC4R genotype (mean±SEM). Weight loss was expressed as a percentage of weight at baseline (surgery). Carriers and non-carriers were matched for age, sex, weight and surgery procedure (gastric banding or bypass). A) Weight loss in carriers and non-carriers of functional MC4R mutations. B) Weight loss data in carriers and non-carriers of the allele rs17782313-C. C) Weight loss data in carriers and non-carriers of MC4R polymorphisms Ile251Leu, Val103Ile, D) Weight loss data in carriers and non-carriers of MC4R polymorphism A_178C.

**Figure 2:**
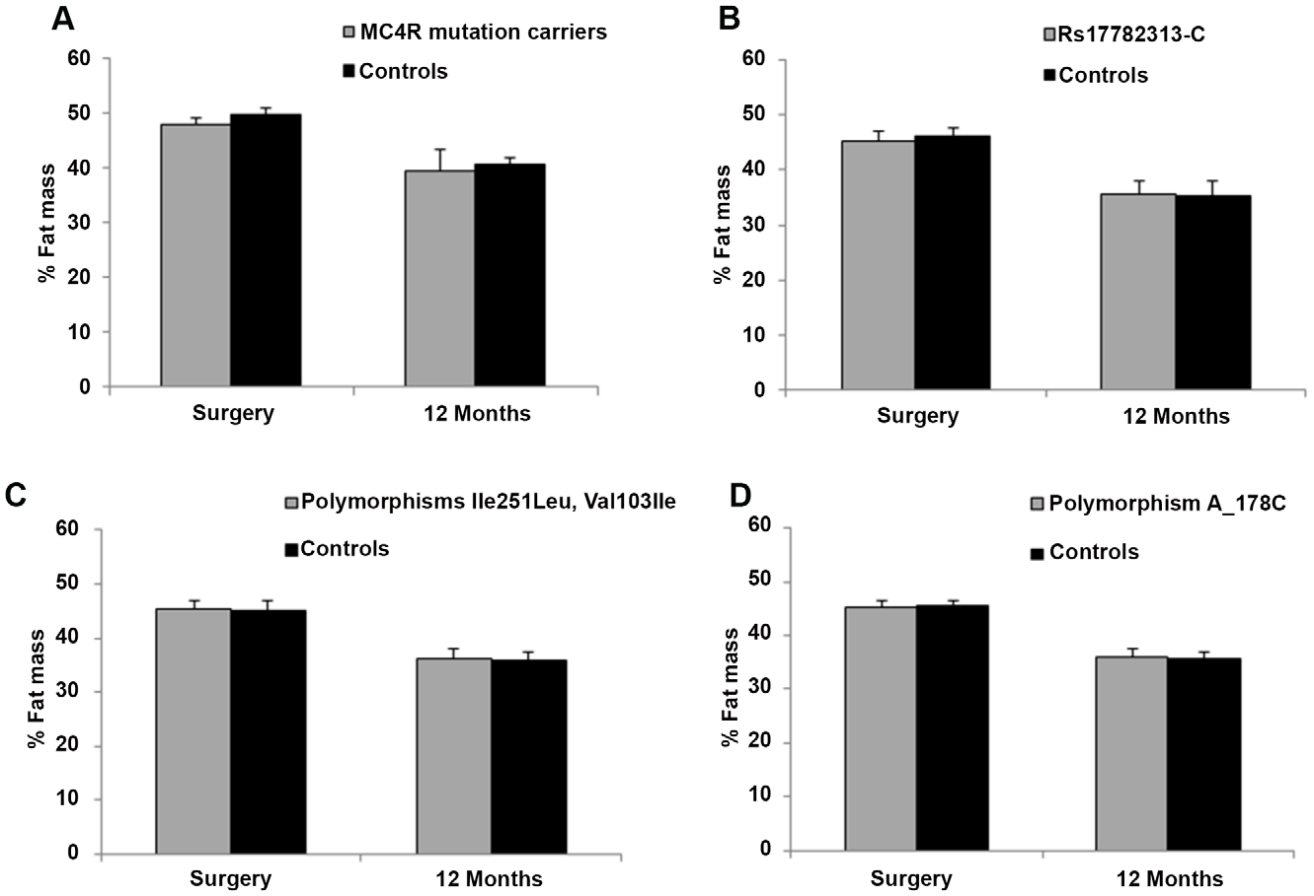
Fat mass percentage at 12 months after bariatric surgery according to MC4R genotype. A, plot percentage of fat mass in carriers and non-carriers of functional MC4R mutations. B, plot percentage of fat mass in carriers and non-carriers of the allele rs17782313-C. C, plot percentage of fat mass in carriers and non-carriers of MC4R polymorphisms: Ile251Leu, Val103Ile. D, plot percentage of fat mass in carriers and non-carriers of MC4R polymorphism: A_178C.

In the other groups, percentage and absolute amount of weight loss (Figure 1) were not related to the presence of *MC4R* mutations. Furthermore, body fat percentages at baseline and after 12 months follow-up were not different between case and controls in each group (Figure 2).

## Discussion

In this case control study we suggest that functional *MC4R* mutations as well as genetic variants (SNPs V103L and I251L, variant rs17782313 and SNP A-178C) did not influence weight loss and body composition after bariatric surgery.

Previous studies in animal models and humans have pointed out the critical importance of the central melanocortinergic pathway in the control of energy homeostasis in particular in the pivotal role of the melanocortin-4 receptor [16]. Based on these findings, we could have expected a different impact on weight following bariatric surgery in human carriers of functional *MC4R* mutations, *MC4R* polymorphisms and variant rs17782313. Our findings extend results of recent observations [17]–[19]. A recent study in 92 morbidly obese adults found similar percentage of weight loss after RYGB between four heterozygous functional *MC4R* mutation carriers and their 8 matched controls [17]. Another study showed similar effects in 15 heterozygous carriers of functional *MC4R* mutations compared to 869 non carriers [18]. In contrary, a case report of a patient who underwent AGB and truncal vagotomy at 18 years of age, suggested that complete MC4R deficiency impairs results in poor weight-loss [20]. This result based on only one patient carrier of two mutations with different effects (a functional MC4R mutation and the SNP I251L) was inconclusive.

In 1443 patients with a minor allele frequency (MAF) of 0.27, the variant rs17782313 located downstream the MC4R gene, presumably associated with increased obesity risk [10], was not shown to be related to maximal weight loss nor weight regain after bariatric surgery [19]. Recent analysis of SNPs V103L and I251L, in a cohort of 1443 obese patients, indicated that twenty-six heterozygous carriers of I251L lost significantly more weight (∼7% initial weight) than the thirty-six V103L carriers and non-carriers. However, in this report, cases and controls were not matched [21], which makes the comparison difficult between groups. Another study in 18 morbidly obese heterozygous for I251L and twenty subjects heterozygous for V103L, did not show any difference after RYGB [18]. Moreover, in a study of 175 patients including 9 carriers who underwent a gastric bypass surgery, MC4R SNPs did not affect weight excess loss, after 18 month of follow up [22]. Concerning polymorphism A-178C in the *MC4R* promoter, its effect on RYGBP outcomes was not yet studied. Our study did not find a consistent effect of this SNP in bariatric surgery outcomes.

Among the four previous published reports only one [17] was designed as a case-control study. However, it was based on a small sample size (four cases and eight controls) which limits its statistical power. Other studies were case series without appropriate matching between cases and controls [18], [19], [21].

Limitations of our study include the rarity of *MC4R* mutation carriers and the short period of follow-up. The strengths are a large group of *MC4R* mutation carriers, separated analyses done according to their functionality and location and the investigation of the impact of bariatric surgery on both weight and body composition. At last, selection bias was limited since controls were randomly selected before matching to cases.

## Conclusions

In conclusion, our study provides good evidence that heterozygous mutations near and in the MC4R gene, either leading to a reduced receptor function or not, did not affect weight loss and body fat mass after bariatric surgery. To have recourse to bariatric surgery as a treatment of severe obesity should not be influenced by *MC4R* mutations status.

## References

[1] Buchwald H, Oien D (2009) Metabolic/bariatric surgery worldwide 2008. Obes Surg 19: 1605–1611. 19885707 10.1007/s11695-009-0014-5

[2] Prospective Studies Collaboration (2009) Body-mass index and cause-specific mortality in 900000 adults: collaborative analyses of 57 prospective studies. Lancet 373: 1083–1096. 19299006 10.1016/S0140-6736(09)60318-4 PMC2662372

[3] Buchwald H, Avidor Y, Braunwald E, Jensen MD, Pories W, et al (2004) Bariatric surgery. JAMA 292: 1724–1737. 15479938 10.1001/jama.292.14.1724

[4] Sjostrom L, Narbro K, Sjostrom CD, Joswiak ML, Karason K, et al (2007) Effects of bariatric surgery on mortality in swedish obese subjects. N Engl J Med 357: 741–752. 17715408 10.1056/NEJMoa066254

[5] Ranadive SA, Vaisse C (2008) Lessons from extreme human obesity: monogenic disorders. Endocrinol Metab Clin North Am 37: 733–751. 18775361 10.1016/j.ecl.2008.07.003 PMC5877402

[6] Hatoum IJ, Greenawalt DM, Cotsapas C, Reitman ML, Daly MJ, et al (2011) Heritability of the weight loss response to gastric bypass surgery. J Clin Endocrinol Metab 96: 1630–1633. 10.1210/jc.2011-1130 PMC3200251 21832118

[7] Loos RJF (2011) The genetic epidemiology of melanocortin 4 receptor variants. Eur J Pharmacol 660: 156–164. 21295023 10.1016/j.ejphar.2011.01.033

[8] Garfield AS, Lam DD, Marston OJ, Przydzial MJ, Heisler LK (2009) Role of central melanocortin pathways in energy homeostasis. Trends Endocrinol Metab 20: 203–215. 19541496 10.1016/j.tem.2009.02.002

[9] Farooqi IS, Keogh JM, Yeo GSH, Lank EJ, Cheetham T, et al (2003) Clinical spectrum of obesity and mutations in the melanocortin-4 receptor gene. N Engl J Med 348: 1085–1095. 12646665 10.1056/NEJMoa022050

[10] Loos RJF, Lindgren CM, Li S, Wheeler E, Zhao JH, Prokopenko I, et al (2008) Common variants near MC4R are associated with fat mass, weight and risk of obesity. Nat Genet 40: 768–775. 18454148 10.1038/ng.140 PMC2669167

[11] Tan K, Pogozheva ID, Yeo GSH, Hadaschik D, Keogh JM, et al (2009) Functional characterization and structural modeling of obesity associated mutations in the melanocortin-4 receptor. Endocrinol 150: 114–125. 10.1210/en.2008-0721 PMC2732289 18801902

[12] Geller F, Reichwald K, Dempfle A, Illig T, Vollmert C, et al (2004) Melanocortin-4 receptor gene variant I103 is negatively associated with obesity. Am J Hum Genet 74: 572–581. 14973783 10.1086/382490 PMC1193776

[13] Stutzmann F, Vatin V, Cauchi S, Morandi A, Jouret B, et al (2007) Non-synonymous polymorphisms in melanocortin-4 receptor protect against obesity: the two facets of a Janus obesity gene. Hum Mo Genet 16: 1837–1844. 10.1093/hmg/ddm132 17519222

[14] Valli-Jaakola K, Palvimo JJ, Lipsanen-Nyman M, Salomaa V, Peltonen L, et al (2006) A two-base deletion -439delGC in the melanocortin-4 receptor promoter associated with early-onset obesity. Horm Res 66: 61–69. 16710097 10.1159/000093469

[15] The Longitudinal Assessment of Bariatric Surgery (LABS) Consortium (2009) Perioperative safety in the longitudinal assessment of bariatric surgery. N Eng J Med 361: 445–454. 10.1056/NEJMoa0901836 PMC2854565 19641201

[16] Ellacott KLJ, Cone RD (2004) The central melanocortin system and the integration of short- and long-term regulators of energy homeostasis. Recent Prog Horm Res 59: 395–408. 14749511 10.1210/rp.59.1.395

[17] Aslan IR, Campos GM, Calton MA, Evans DS, Merriman RB, et al (2011) Weight loss after Roux-en-Y gastric bypass in obese patients heterozygous for MC4R mutations. Obes Surg 21: 930–934. 20957447 10.1007/s11695-010-0295-8 PMC3119798

[18] Hatoum IJ, Stylopoulos N, Vanhoose AM, Boyd KL, Yin DP, et al.. (2012) Melanocortin-4 receptor signaling is required for weight loss after gastric bypass surgery. J Clin Endocrinol Metab 97. 10.1210/jc.2011-3432 PMC3387412 22492873

[19] Sarzynski MA, Jacobson P, Rankinen T, Carlsson B, Sjostrom L, et al (2011) Associations of markers in 11 obesity candidate genes with maximal weight loss and weight regain in the SOS bariatric surgery cases. Int J Obes 35: 676–683. 10.1038/ijo.2010.166 20733583

[20] Aslan IR, Ranadive SA, Ersoy BA, Rogers SJ, Lustig RH, et al (2011) Bariatric surgery in a patient with complete MC4R deficiency. Int J Obes 35: 457–461. 10.1038/ijo.2010.168 PMC4648366 20733581

[21] Mirshahi UL, Still CD, Masker KK, Gerhard GS, Carey DJ, et al (2011) The MC4R (I251L) allele is associated with better metabolic status and more weight loss after gastric bypass surgery. J Clin Endocrinol Metab 96: 2088–2096. 10.1210/jc.2011-1549 PMC3232628 21976721

[22] Goergen M, Manzoni D, De Blasi V, Fabiano P, Poulain V, et al (2011) Influence of obesity-susceptibility loci (MC4R and INSIG2) on the outcome of weight loss and amelioration of co-morbidity in obese patients treated by a gastric-bypass. Bull Soc Sci Med Grand Duche Luxemb 2: 7–24. 22272442

